# Abstracts and Gleanings

**Published:** 1880-01-20

**Authors:** 


### ABSTRACTS AND GLEANINGS.

**Lactic Acid in Catarrh of the Bladder.**—Dr. Deecke, in *Buffalo Medical and Surgical Journal*, says: It is about two years ago that my attention was called to the almost constant occurrence of micrococci and gliococci in the urine of patients who suffered from chronic catarrh of the bladder, or from those forms of acute cystitis which were accompanied by an excretion of urine of a neutral or alkaline reaction. This occurrence induced me to a series of experiments with different substances, prominently acids, for the purpose of studying their action upon the development and the growth of those organizations, and of exploring their influence upon the alteration of the liquid which served as their pabulum. The experiments were first made in open test-tubes, and were repeated by excluding the air as much as possible. The pabulum was normal urine and urine of a patient who had been suffering several months from a mild chronic catarrh of the bladder, and who had been treated, as related, for "some form of Bright's disease of the kidneys," the general dread of all who experience some chronic disorder in the uropoetic system. The tried substances were—silicate of soda, sulphite of soda, permanganate of potassa, sulphuric, nitric, and muriatic acid; acetic, citric, and tartaric acid; carbolic acid, salycilic acid, and lactic acid.

As the experiments were of a decided result in one direction, it is of no interest and consequence here to enter into the narration of any details combined with them.

There is among all these substances only *one* the effect of which was found to be beyond comparison with any of the others, and this is *lactic acid.* An addition of one per cent. of lactic acid prevents a decomposition and alkaline fermentation of normal urine, and the development of micrococci and gliococci for a long period; in pathological urine it arrests their growth and multiplication almost instantly. It seems to be a specific poison for these microscopic forms of life.

As the antiseptic properties of lactic acid have been known for years, it is the more surprising that it has not been tried long ago in these special cases, since all remedies hitherto recommended, according to the experience of the latest expert, Dr. Max Schuller, of Griefswald, "only met the one or the other demand with the desired effect."

It may be remarked here at once that the antiseptic action of lactic acid, by local application, is by far not the only effectual property of this drug. Its dissolving power for catarrhal and diphtheritic exudations is likewise known, and should not be undervalued; it is superior to that of any other acid. It furthermore dissolves easily the ammonium compounds, the ammonio-triple phosphates and the calcium phosphates. And best of all, it permits also of an internal administration in a most agreeable form, as a kind of lemonade, or in the form of buttermilk or added to it. It will be discovered in the excreted urine, after a few good doses, a fact which was established by a number of careful analyses of the urine after internal administration of the acid. This generally occurs when three or four grammes of the drug have been consumed—an effect which will be accelerated by a cotemporary use of buttermilk as a beverage. The latter property of course makes the remedy the more valuable, especially in all acute cases and those of a milder course, or where the condition of the patient or other circumstances are not favorable to a local treatment. As to the administration of the drug, the acid may be given in doses of 1 to 1.5, even to 2 grammes three times a day in sugar and water. Even the large doses administered in a diluted form, or in a mild bitter tonic, when continued for a longer period, do not interfere with digestion. A hypnotic influence has not been observed. For local treatment a solution of 1.5 to 1 per cent. will suffice, and I recommend two injections at one session for the beginning, with the cotemporary internal use of the acid, and not to repeat the injection sooner than absolutely necessary. In the majority of cases, even of long standing, only a few injections will be required.

As to the instruments for injecting into the bladder, a simple silver catheter is preferred to those with a double tube. For a syringe I recommend a fountain syringe or syphon syringe (like the common nasal douches) with a rubber tube about three feet in length. If the catheter is provided with a stop-cock at its base part, or with a short rubber tube closed by a clamp, it can be introduced filled with the injecting fluid, and the connections can be made with the fountain tube in such a manner that all air is excluded inside of the apparatus, which will be found of great convenience to the patient, as well as to the attending physician. The manipulations are simple. The tumbler containing the injecting fluid, the temperature of which should be raised to about 100 degrees Fahrenheit, is placed on the floor, the tubes are filled with the fluid, and the connections are made with the catheter in position, which must be supported by the left hand or by the patient himself. The right hand then raises the tumbler from the floor, slowly, to the desired height. The bladder fills up by this arrangement without any inconvenience to the patient, or any sudden shock, and the pressure from the column of injecting fluid can be entirely controlled, and the bladder can be expanded ad libitum.

During about eighteen months twenty-one cases have been under treatment, of which a record was made, for the most part by practical physicians of my acquaintance. Among these there is only one case to be mentioned of a chronic catarrh of the bladder from a stricture in which the recovery remained doubtful because of an interruption in the treatment. In six acute cases (5 male and 1 female) the cystitis had originated from an inflammatory condition of the urethra; in only one of these it was found necessary to have recourse to a twice repeated injection into the bladder. Five acute cases developed without any known cause, recovered rapidly from an internal use of the acid. One case of a chronic catarrh from partial paralysis of the bladder required three double injections; one case of hypertrophy of the prostate four injections. From eight cases of chronic catarrh of long standing only in one the injection was repeated ten times, three recovered from the internal use only, and the remaining four cases submitted to two, to three and four injections.

**Meniere's Disease.**—Dr. Gu*g*e, in British Medical Journal, says: 1. In a general sense of the word the name of Meniere's disease may be applied to all cases of vertigo which are caused by an abnormal irritation of the nerves of the semi-circular canals. The irritation may be produced either by an exaggerated normal cause, as violent rotary movements, or by an abnormal cause, as a sudden change of temperature, variations in the intra-tympanic pressure, disturbances in the circulation, or inflammation.

2. In a more restricted sense, the name Meniere's disease is applied to cases where the vertigo is caused by an inflammatory condition either of thesemi-circular canals or of the middle ear. The vertigo may be either persistent or simply momentary by normal movements of the head. In some cases it appears periodically under the form of a fit at intervals of weeks, or even months.

3. Exposure to cold and catarrhs of the tympanic cavity play a prominent part in the etiology of Meniere's disease.

4. The majority, if not all, cases are secondary to catarrhs or inflammations of the tympanic or mastoid cavity.

5. In typical cases the vertigo is preceded or accompanied by rotary sensations which follow a certain order; the attack begins by a sensation of rotation around a vertical axis. The rotation invariably takes place on the affected side; sometimes it is combined with a sensation of swinging backwards and forwards. In more serious cases the feeling is that of rotating round a horizontal axis, both backwards and forwards. Finally the vertigo becomes general and the patient loses consciousness and falls down; he often vomits in such cases. Sometimes the attack is over in from ten to thirty minutes, in other cases it is called forth by a simple movement of the head during one or two days following the attack, and the patient is obliged to lie perfectly still in order to avoid them.

6. In some cases the rotary sensation may be caused experimentally by certain therapeutic agents, as the insufflation of air into the tympanic cavity in cases of acute inflammation of the latter. In these cases the rotary sensation always takes place round a vertical axis and in the direction of the affected organ.

7. In some cases the attacks are accompanied by loud noises in the ear; in other cases there is a constant slight buzzing noise, which does not increase in strength during the attack; sometimes there is no noise at all.

8. In cases of long standing a slight feeling of vertigo persists even during the free intervals, and seems to be caused by the first movements of the head after waking from sleep. Some patients are obliged to keep the head fixed in a certain position, because every movement that takes place in the plane of one of the semi-circular ducts is accompanied by a sensation of a heavy body rolling in the same direction.

9. Meniere's disease is frequently complicated with hysteria. It is also apt to produce in children a condition not unlike chorea, and in adults clonic contractions of muscle of the face and body. These often disappear after local treatment of the middle ear.

10. In some cases patients, after recovering from Meniere's disease, have lost the faculty of hearing.

11. Highly satisfactory results have often been obtained by local treatment even in inveterate cases.—*Detroit Lancet.*

**Little Things in Obstetrics.**—Shall the patient be purged the first, second, or third day, and debilitated in this way? I think not; indeed, I am sure that it is a bad practice. If the patient's bowels have been kept regular before confinement, and moved the day of delivery, there is no necessity of disturbing them the first two days, or even the third day, and then they must be moved by enema. Of course there are cases where there will be special reasons for opening the bowels with medicine.

Speaking of bowels, I like the old plan of having the rectum evacuated before delivery, and if the bowels have not been moved that day an enema of warm water may be used to evacuate them when the first pains are felt. I recall a case in the early part of my practice in which labor progressed in the usual way until the head passed down into the pelvic cavity, when it met an obstruction that prevented a further advance. The finger in the vagina felt a hard mass in the lower part of the hollow of the sacrum, which might be an exostosis, or a fibroid tumor, but which evidently closed the outlet of the pelvis, so that the child could not pass. There was doubt about what it might be for some time, until finally the thought occurred that it might be impacted feces, which was soon proven by a finger introduced into the rectum. It required a full half hour's hard work to break down the accumulation and bring it away. In another case I recall, the large and rough fecal mass was forced through the anus by the advancing head of the child, with the effect of producing a very painful laceration. In this case the physician came in just as the head was passing.

I do not see the necessity of a woman's suffering from after-pains, and stlll less the propriety of giving narcotics to relieve them. Macrotys or viburnum will relieve them, and I usually combine a very small portion of aconite with the one or the other to prevent fever. After the second child I always leave for the patient, in case of after-pains, a prescription something as follows:

℞. **Tinct. Aconite gtt. v.,**

**Tinct. Macrotys, gtt. xx.,**

**Water..................℥ iv.**

A teaspoonful as often as required.

Milk-fever is always looked for and provided against. A small dose of aconite with phytolacca to meet any irritation of the breasts, will meet the indications, and be a source of such comfort that the physician in attendance should not forget it. Again, phytolacca is a most excellent remedy when there are any indications of nursing sore mouth, and will cure a large proportion of cases if the disease is taken at its commencement.

Very severe pain, recurring frequently or almost constant, the first, second, or third day, should suggest that a fragment of the placenta or a portion of the membranes, was engaged in the os. When I was a very young obstetrician, I had a case of this kind, and the pain was so intense and prolonged that I called counsel. A very small piece of placenta was removed, and the pain stopped. I was called in one case to see a woman who had been delivered by a midwife, and who had suffered most excrutiating pain for twenty-four hours. The piece of placenta engaged in the os would not have weighed more than half an ounce, and with its removal the pain ceased.

A fetid lochial discharge is a very unpleasant thing, and it may give rise to irritation or puerperal fever, or be a cause of local disease. I have never been able to see why a woman should remain dirty after delivery, the unpleasant secretions drying about the vulva and nates, and accumulating in the vagina. It seems to me that there is a necessity for soap and water here and thorough cleanliness, if there are any unpleasant discharges. Whilst an injection of chlorate of potash water may be carefully used in the worst cases, sponging the parts will be sufficient in the majority.

Whenever there is marked fetor, I prescribe chlorate of potash internally in the usual doses. I have little fear of pyæmia, or puerperal fever from absorption, when this is taken.—*Eclectic Medical Journal.*

**The Value of Calomel in the Zymotic Diseases of Infancy.**—Dr. E. M. Boddy expresses his views as follows, in the *Medical Press and Circular*, October 8th:

I shall make a few remarks on the advisability of administering calomel in diseases which are specially peculiar to infancy, such as scarlet fever, measles, and others of a zymotic type.

In all the zymotic or exanthematous fevers, there is the accompanying eruption or rash, as it is usually called, which, when it has thoroughly exhausted itself, or in other words, when it has finally disappeared, and the desquamation of the cuticle has commenced, then is the time to direct our attention to the alimentary canal, for we shall invariably find after, as well as during the attack, that the alvine excreta are in a most filthy and unhealthy condition, in fact, almost approaching a poisonous character, and, as some believe, contain an element highly infectious to the last degree, and especially when the patient is suffering from typhoid fever. Regarding these infectious or non-infectious characteristics, I have nothing to do; but, parenthetically, I may say, they develop gases, exceedingly offensive and injurious if inanadvertently inhaled; they must, therefore, be extremely detrimental to the recovery of the sufferer, for if they are poisonous when ejected or exposed to atmospheric influences, what must they be when allowed to remain in the intestines, pent up in a confined space, with the mucous membrane absorbing the impurities resulting from the effects of the fever, besides the impure liquid portion of the fæces; what must be the result, I say—a protracted recovery or a certain death?

Therefore, it behooves us, immediately on the disappearance of the rash, to administer purgatives till we have eliminated the fever poison which has been germinating and stagnating in the fecal contents of the intestinal canal, and the only purgative which is at all capable of thoroughly cleansing out the intestines is calomel; for, owing to its dual properties, it not only purges the patient, but by virtue of its chologogic action, it cleanses out the human cess-pool, viz: the liver, which, in all fevers, is a reservoir for everything impure and unhealthy.

If we do not pursue this course, the inevitable result is diarrhœa, which, instead of being regarded as a good omen, as indicating that nature requires assistance, and that she is trying to accommodate herself to the force of circumstances, we go diametrically opposite to her, and regard the efforts of nature as significant of approaching evil; and so we resort instanter to astringents, and if that is not sufficient (and it very seldom is), we inject up the rectum certain astringent compounds, which is as unscientific as the insertion of a cork would be; we know or can guess the result—the child dies, presumably from the fever, though I cannot help thinking that the child succumbs to the deleterious action of the astringents.—*Medical Reporter*.

**Effects of Local Irritation on Pain.**—At the meeting of the Academie de Medicine on the 4th of November (*Bulletin*), Dr. Dumontpallier read a memoir on Local Therapeutical Analgesia induced by the Irritation of the Similar Region on the Opposite Side of the Body.

"From this communication it results that pain seated at one point of the body yields to an injection of simple water (which, as is known, produces local irritation) at a similar point on the opposite side. In neuralgias of different seat and nature, in acute articular rheumatism, and in rheumatic or toxical neuralgia, I have requested patients to mark with the finger the painful points, and that being done, I have sought out similar points on the opposite side of the body, and at these latter points, for the most part not painful, I have practiced injections of water or simple punctures. As soon as irritation has been produced on the sound side, the patients have acknowledged a diminution, and often a complete cessation, of the pain on the bad side, and that, I repeat, in cases of acute rheumatic arthritis. I have chosen this last example as a demonstration, as one could scarcely in such a case be deceived by patients. The joint may be red, swollen, hot and painful to the touch or the slightest movement, but immediately the little operation is terminated, the patients find that the pain diminishes or disappears, and that they can perform flexion or extension of the joint; the swelling preventing much motion, but the pain is gone."

The following are the conclusions arrived at by Dr. Dumontpallier: 1. Every subcutaneous medicinal injection is a complex operation, in which a part must be assigned to the medicinal substance, and a part to the irritation produced. 2. The local irritarion is transmitted from the periphery to the sensitive centres, and there determines a modification, the consequence of which is a diminution or cessation of the peripheric pain. 3. The real, anatomical seat of certain peripheric pains should then be in the sensitive centres; an assertion which seems demonstrated by the crossed action of induced peripheric irritation. 4. Irritation induced loco dolenti, or in the vicinity of the painful point, assauges or causes the cessation of pain; and when the irritation is induced at symmetrical points on the opposite side of the body, it proves often sufficient to cause a complete and durable cessation of pain.—*Medical Times and Gazette*.

**Rules for the Treatment of Croup.**—The following rules are laid down by Dr. W. H. Day, as the result of a long experience in this disease (Medical Press and Circular, November 5th, 1879):

The temperature of the room should not be lower than 65 degrees.

1. The vapor bath is indispensable in the treatment of croup, and should be used at the commencement in every case, and continued unremittingly until all fear of a relapse has departed.

2. All cases of croup are invariably relieved by the vapor bath, especially if the tracheal membrane is dry; when it is moist there might be fear of causing too much depression.

3. The earlier that a case comes under treatment, the greater the probability of a successful termination, because it is then possible to prevent the tracheal secretion becoming organized.

4. The most trying difficulty we have to contend with in the management of croup in the catarrhal form is a relapse, because with it comes exhaustion; and the weaker the patient the less will be the chance of recovery.

5. Tartarized antimony should, however, be mainly given for the purpose of producing vomiting; that failing, it is comparatively useless, because, if continued in small doses at intervals, its depressing effect is too great.

7. When the emetic has fully operated, if there be much febrile excitement and disordered primæ viæ, which aggravate the laryngeal symptoms, a grain of calomel every four hours. or one full dose for the purpose of emptying the bowels and controlling the fever, will be found necessary. In the fibrinous form, when there is violent and acute inflammation, with a firm, hard pulse, and a full reserve of strength, two or three leeches may be applied over the thyroid cartilage, and bleeding can easily be arrested by pressure with the finger, and if need be, with cotton wool; then mercury may prove a valuable addition to the antimonial treatment. Some of my cases improved from the moment the mercury affected the bowels, the fever diminishing, and the expectoration of the false membrane being promoted. When employed in small doses at regular intervals it would appear to diminish the cohesive attachment to the mucous membrane, and to render the lymph less fibrinous and more readily absorbed.

8. When in a case of croup, seen at an early stage, and satisfactorily progressing, forty-eight hours have elapsed, we may generally augur a favorable termination; and we should then begin, if not before, to support our patients with good beef tea, milk and arrowroot, and (it may be) a little wine and water.

If after vomiting the temperature remains high, and especially when the bowels have acted freely, minim doses of aconite every two or three hours are of great service in inflammatory croup. This keeps up a gentle diaphoretic action on the skin, diminishes tension of the pulse, and controls vascular excitement in a very striking manner. At this stage it comes in well, because antimony should not be long continued in any of the diseases of children, and it certainly ought not to be in this disorder.—*Medical and Surgical Reporter.*

**Treatment of Fibroid Tumors of the Uterus by Ergotine Suppositories.**—Robert Bell, M.D., F.F.P.S.G., Physician to the Glasgow Institution for Diseases of Women and Children, reports the following:

The immense benefit resulting from the subcutaneous injection of ergotine in uterine fibroids has been so often demonstrated that we now look upon the drug as the most potent agent we have in the medical treatment of such tumors, and yet there are many objections to its use by means of the hypodermic syringe. First among these is the tendency to suppuration in the neighborhood of the acupuncture, and when the operation requires to be repeated twice a week this is no small disadvantage. True it has been said that if the needle is insinuated into the muscular tissue, this evil result is avoided, but I must confess this has not been my experience. Then the operation is attended with an amount of pain which is not inconsiderable, and when we bear in mind that the subject of uterine diseases is generally in a high nervous state, this resulting from the fact that her uterus is diseased, there is as a consequence a great repugnance on the part of the patient to undergo, what to her sensitive nature is magnified, an operation twice a week, with its sequela of an abscess in each instance. To avoid the unpleasantness of using ergotine hypodermically, I have for some years had recourse to suppositories, each containing four grains of the drug; one of which is introduced every night; and, with a view of illustrating the beneficial effects of the remedy used in this way, I have taken cases at hap-hazard, one of which I will briefly record:

CASE.—Mrs. G——Saltcoats, Ayrshire, sent for me in May, 1875. She was suffering from what appeared to me on a hurried examination to be acute retroflexion of the uterus, but on passing the sound it became quite evident that what I had at first mistaken for the fundus of the uterus was a fibroid tumor. The uterus was lying in its normal position; was not more than the usual length, but it was exquisitely sensitive; and on the posterior aspect there was, protruding towards the rectum, an interstitial fibroid occupying the posterior wall of the organ. The presence of the tumor, taken along with the metritis which accompanied it, had induced great prostration of body, and a low morbid condition of the mind of the patient. To such an extent had the weakness proceeded that the lady was unable to leave her bed for more than a few minutes at a time, and walking always brought on excrutiating pain after a few steps had been taken. She was ordered suppositories, containing two grains of ergotine in each, one to be introduced every night. In three weeks she was able to walk about with considerable freedom, and in two months was able to come to Glasgow, when I found the tumor was very much reduced in size, and the general health of the patient exceedingly good. This was the first case in which I had given a fair trial to the ergotine suppositories; but it was a matter of regret to me that I had not used four grains instead of two grains in each. Latterly the strength of the suppository has been doubled, and I find that the result is not only more speedy, but much more satisfactory in every way.

I would urge all who employ ergotine in such cases, or indeed in any disease, to be sure that they are being supplied with a good article, or, I may hardly add, disappointment will most certainly follow. This caution is the more necessary, as ergot of rye is often supplied to the trade, which, when examined, is found to be absolutety useless as a medicinal agent. We cannot, therefore, be too careful in our selection of the drug, nor too particular in our investigation of the efficiency of each individual sample.—*Obstetric Gazette.*

**Popular Nostrums.**—*Walker's California Vegetable Vinegar Bitters.*—Each bottle contains from nineteen to twenty fluid ounces, consisting of a decoction of aloes and a little gum guaiac, anise seed and sassafras bark, in water slightly acidulated with acetic acid, possibly the result of secondary fermentation, or added in the form of sour cider. Each bottle contains also about one ounce of Glauber's salt, one-quarter of an ounce of gum arabic, and from one-half to one ounce of alcohol.

*Radway's Ready Relief*—This is a light brown liquid, consisting of eight parts of soap liniment, one part of the tincture of capsicum, and one part of aqua ammonia.

*Radway's Renovating Resolvent.*—Each bottle contains six fluid ounces of a vinous tincture of cardamon and ginger sweetened with sugar.

*Pierce's Golden Medical Discovery.*—Each bottle contains one drachm of the extract of lettuce, one ounce of honey, one-half drachm of the tincture of opium, three ounces of dilute alcohol, and three ounces of water.

*Pierce's Favorite Prescription.*—A greenish-brown turbid liquid, consisting of a solution of one-half ounce of sugar, one drachm of gum arabic, in eight ounces of a decoction made from two drachms of white agaric, one and one-quarter drachms of cinnamon, and two drachms of cinchona bark; to this mixture are added one-half drachm of tincture of digitalis, and a solution of eight drops of oil of anise in one and one-half ounces of alcohol.

*Van Buskirk's Fragrant Sozodont.*—A red liquid consisting of a solution of one-half drachm of white castile soap in one ounce of water, and one-quarter of an ounce of glycerine, colored with cochineal, and flavored with oils of winter-green, cloves and peppermint. The powder which accompanies each bottle consists of a mixture of precipitated chalk, powdered orris root and carbonate of magnesia.

The above are taken from Hoffman's "Popular Health Almanac," a publication which is meant to serve as an antidote to the numerous almanacs distributed broadcast through the country as a means of advertising various patent nostrums.

*Ayer's Cherry Pectoral.*—

| | | | |
|---|---|---|---|
| ℞ | Morph. acetas.......................................... | gr. iij. | |
| | Tr. sanguin. canaden.................................. | ℥ ij. | |
| | Vini antim. et potas. tart.............................. | | |
| | Vini ipecac............................................aa | ℥ iij. | |
| | Syr. pruni Virgin....................................... | ℥ iij. | M. |

—*Hospital Gazette.*

**Surgical Treatment of Goitre.**—Dr. Wolfer, in speaking of the treatment of goitre with subcutaneous injections of iodine, says, in Langenbeck's Archives, that favorable results have been obtained both in cases of simple hyperplasia, and of colloid degeneration. He illustrates his statements by a few cases from Billroth's clinic, and an experiment on a dog, made by himself.

The lobes of the thyroid gland of this dog had respectively attained the size of a goose's egg, and the author made ten injections of iodine into one of the lobes. The dog was killed at the end of a month, when the portion of the goitre into which the injections had been made was found to have dwindled down to the size of a man's thumb; it consisted of connective tissue which no longer contained any colloid liquid. The peripheric part of the injected goitre presented the same appearance as the lobe which had remained untouched; it consisted of large meshes of connective tissue, which contained colloid fluid. There were no traces of inflammation or hemorrhage following the injection of iodine.

Several strumous cysts were treated in a different manner: One cyst with thin walls was absorbed after injections of iodine; two other cysts resisted this treatment. In two cases Billroth drained strumous cysts with antiseptic precautions. In one of these cases, the cure was speedily effected; in the other, the cyst was not wholly absorbed, as there were calcareous deposits in its walls. The sac was then opened and the contents removed, after which the patient, a woman aged 72, recovered.

The author thinks that tapping the cyst and putting in a drainage tube ought to be done in cases where a cyst does not collapse immediately after being tapped, or in old people where the injection of iodine might be succeeded by a too strong reaction, but where extirpation of the goitre might prove fatal. In the course of the last year, Billroth has extirpated goitres in seven cases under antiseptic precautions, the results having each time been very favorable. In one of these cases the patient was suffering from malignant cystous papilloma; in another case the struma was of carcinomatous nature. All the wounds healed by first intention.—*London Med. Record.*

**Unusual Effect of a Hypodermic Injection of Morphia.**—Dr. Aug. M. Tupper, of Rockport, Mass., reports, in the Boston Med. and Surg. Journal, the case of a healthy-looking man, aged about 30, who was suffering severely from lumbago; counter-irritants having failed to afford relief, he injected directly over the seat of pain nine drops of a solution of sulphate of morphia, one grain to a drachm of water with one drop of carbolic acid to keep it. In five minutes the patient was relieved. In about five minutes later he complained of nausea; before a basin could be given him he grew deadly pale, his eyes rolled up, so that only the whites were visible, jaws were clenched, head drawn back, the whole body stiffened, respiration ceased, and the pulse at the wrist was absent. Cold water was instantly dashed in his face. In about a minute his eyes were observed to be widely open and staring, and the pupils widely dilated. Very soon the color began to return to his face, he was drenched with perspiration, and recovered consciousness. His pulse was now sixty, full, but irregular.

Dr. Tupper has given the same mixture a great many times without the slightest trouble. The solution was freshly prepared the morning it was used, and the same dose had been, on the same day, injected under the skin of a neuralgic female before using it on the patient whose case is above narrated. The injection entirely relieved the lumbago.

This and similar cases which have been reported from time to time show that in patients whose tolerance to the hypodermic use of morphia is not known, it is not safe to begin with a larger dose than one-eighth of a grain.—*Med. News.*

**Treatment of the Fibrous Tumors of the Uterus.**—At the late International Medical Congress at Amsterdam, Dr. J. De La Faille read a paper on this subject, of which the following are the conclusions:

1. The mode of treatment of fibroid tumors of the womb depends principally upon the flow of blood that accompanies them.
2. The seat of the tumors and their development modify the treatment.
3. Internal medication offers but little prospect of success, though it may be tried in intra-parietal fibromas. The same may be said of alkaline baths.
4. One of the most rational modes of treatment of intra-parietal fibromas is that of subcutaneous injection of ergotine.
5. The plan of dilating the womb by means of the prepared sponge of laminaria is not without danger; it requires at least a prompt renewal of the dilating substances.
6. Linear ecrasement is preferable to any other method for operating upon fibrous polyps.
7. Intra-uterine fibromas are best removed by enucleation. The same applies to sub-peritoneal fibromas.
8. In cases of gastro-hysterotomy, intra-peritoneal treatment of the pedicle is preferable to extra-peritoneal treatment.
9. Total extirpation of the uterus offers some great advantages.
10. Castration is seldom indicated in cases of fibrous tumors of the womb.—*Arch. Gen. de Medecine.*

**Intra-Tympanic Injections.**—Dr. Weber Liel, in *British Med. Journal*, says, that sixteen years of experience in aural practice has forced him to give up the idea that it might be possible to cure inveterate catarrh of the tympanic cavity by means of intra-tympanic injections of medicated fluids.

1. The symptoms of catarrh of the tympanum may depend upon extension of a simple catarrh from the eustachian tube and the pharyngo-nasal cavity; then the latter only must be the object of treatment. In this treatment injections of strong nitrate of silver solutions into the mouth of the eustachian tube, followed four days afterwards by the use of the air douche, will be found to have the best effects in reducing the catarrhal symptoms. But, in order to avoid inflammation of the tympanum, not more than a few drops of the solution must be blown in with force by means of the eustachian catheter; and the patient must be forbidden to blow his nose till four hours after the injection.
2. The symptoms of the intra-tympanic catarrh are due not only to a catarrh of the tube, but to a collapse of the walls of the eustachian canal dependent on insufficient or paralyzed action of the eustachian tube muscles. In such cases, not intra-tympanic injections, but the awakening of the activity in the tubal muscles, by intra-tubal electricity, must be the treatment to cause the disappearance of the symptoms of the secondary intra-tympanic vascular stasis and catarrh.
3. Symptoms of congestion and catarrh of the tympanic cavity may arise from alterations of the vaso-motor and trophic nerves and of the sympathetic supplying the tympanic cavity. Solutions of carbonate of soda diminished mucus, incrusted and transuded purulent matter and softened adhesion.—*Detroit Lancet.*

**Traumatic Tetanus.**—Dr. Molliere relates the following case in the Gazette des Hopitaux. The patient, aged 25, had been accidentally shot in the right foot. The fourth and fifth toes were so badly injured that they were amputated at once; the first phalanx of the third was fractured, and the articulation opened, but it was thought that it might be preserved.

The patient was treated antiseptically, and seemed to progress well during a fortnight, when suddenly he began to complain of a feeling of lassitude, the wound became very painful, and he experienced some difficulty in opening his jaws and turning his head. The toes were dressed with laudanum, and the patient took half a drachm of bromide of potassium and a drachm and a half of chloral daily; he had also two hypodermic injections of morphia.

Notwithstanding this treatment, the patient became worse, the pain in the foot increased, and all the symptoms of acute tetanus showed themselves; he had general convulsions, could not move his head or open his mouth, perspired abundantly, had very high temperature, etc. The wound becoming exceedingly painful, the injured toe was amputated.

From that day the local pain ceased, and the other symptoms gradually vanished. The patient remained sleepless for a rather long time, notwithstanding the use of hypnotics, but could open his mouth freely, and could swallow. Smaller doses of chloral and bromide of potassium were given, and a month after the operation the patient was well enough to leave the hospital.

On dissecting the toe which had been removed, it was found that a small sharp fragment of bone was sticking in the internal lateral nerve, and had in this way caused the tetanic convulsions.

This case is remarkable on account of the different methods of treating tetanus having been combined in the treatment. Without the amputation, the drugs given would have had no effect; but on the other hand, if the powerful doses of hypnotics had not been administered, the surgical treatment would, in the author's opinion, have proved useless.—*British Med. Journal.*

**The Method Used in Germany in the Treatment of Placenta Prævia.**—The earliest possible rupture of the membrane must be the safest check to the bleeding, and, indeed, this treatment has had the best results. It is a mistake to suppose that the bleeding can be stopped by compression of the presenting portion. In a few of these cases treated in the lying-in hospital, the bleeding ceased immediately on rupturing the membrane, although only a thigh lay in the orificium uteri, which did not at all fill it out. A forced birth is, however, not advisable, as the cervix in placenta prævia, although it may be dilated, is also easily ruptured.

Dr. Bennicke has, in nine out of twelve cases treated in the city, ruptured the membrane early; then by a combined version pulled down a foot, and then allowed nature to finish the delivery. The twelve women lived, also four of the children.

Dr. A. Martin ended forty-one cases operatively, and lately has advocated as the safest the treatment herein prescribed. He recommended the rupture of the membranes because the disadvantage of the tampon, especially the infection, is thereby avoided. The cervix is not always easily dilated; for instance, he once saw a case of rupture occurring during the passage of the head, and by which the woman bled to death. He tried, only once, Kleinwaechter's method to loosen the placenta considerably, and then to inject ice water, and with bad result.

After the birth of the child the placenta must be immediately removed. Against post-partum hemorrhage he recommends injection of hot water in the uterus.

Dr. Jacquet expresses himself also in favor of early rupture of the membrane and of combined version.—*Buffalo Medical and Surgical Journal.*

**Tooth Brushes.**—Dr. Palmer, in Dental Cosmos, says:

"Concerning the forms of brushes, I will say that straight brushes are utterly impracticable on the surfaces to which I have referred as the ones most neglected. Curved brushes with a tuft end, bud-shaped or convex, are the best.

"There are several favored forms that are quite efficient in the line I have spoken of. One of these, named the 'Windsor,' I have faithfully tried for twenty months past, and introduced it very generally in my practice, and I feel that it meets the indications better than any other within my knowledge. The faithful use of floss-silk between the teeth ought to be earnestly recommended; also the *quill* toothpick. The wood toothpicks so generally furnished at public eating-places are a source of much evil to the soft tissues between the teeth. All kinds of metallic toothpicks are objectionable, though I am aware that it is the practice of some dentists to commend them to their patients."

**The Metric System.**—The metric system does not seem to be making great headway among medical men in this country, but perhaps the progress is as grod as could be fairly expected. At present decimal fractions are less familiar than common or "vulgar" fractions to druggists, as to most other business people, and practice alone can give expertness and accuracy in the use of the former. We suspect that this is really one of the chief obstacles to the general introduction of the metric system. To the popular mind ½, ¼, ⅛, etc., are symbols that are instantly understood, while the corresponding decimals .5, .25, .125, etc., are but slowly apprehended. When the fractions are less simple, the difficulty is proportionately greater. Our familiarity with Federal money does not help us here, for in that we use no decimals beyond hundredths (we write .12½, not .125, etc.), and read them as *cents*, not as fractions of a dollar.—*Boston Journal of Chemistry.*

**A Daring Operation.**—An operation was recently performed by Pean, of Paris, which for boldness is perhaps unique. The patient was suffering from cancer of the pyloric extremity of the stomach, completely blocking up the passage. He removed the pylorus and stitched the severed end of the stomach to the duodenum. The patient died on the fifth day.

**Gall Stones.**—Dr. Buckler, in an article in the Boston Medical and Surgical Journal, seems to place the utmost confidence in the use of chloroform to dissolve gall stones. He gives 15 to 20 drops, or more, every three or four hours, and claims to accomplish solution in a week or ten days.

In cases where the use of chloroform is objectionable, and in all cases of the use of chloroform, he uses the succinate of iron, in doses of half a teaspoonful after each meal. He says: Of all the certainties of medicine, there is nothing more absolutely sure than that chloroform will in every instance dissolve calculi in the gall bladder."—*Ohio Med. Recorder.*

**Hypodermic Injections of Ether in Sciatica.**—In a case reported in Lancet & Clinic, by Drs. Whitaker and Maris, the first injection of fifteen minims immediately produced warmth and reduced pain, and patient slept soundly that night for the first in two weeks. Afterwards used five injections on the five days succeeding, one each day, averaging twenty-five minims each. Improvement continued steadily. Patient put on comp. phos. pil. and iron. Appetite immediately returned, sleep returned; use of leg gradually returned, muscular atrophy disappearing, and he is now well. No abscess formed, only slight callous forming around the puncture.

**Propylamine in Acute Rheumatism.**—Dr. Gaston, of Indiana, says that this agent will subdue the pain in 24 to 48 hours. Dr. Tyson, of Philadelphia, also recommends it where the salicylate of sodium is for any reason inapplicable. His formula is:

℞. Proplylamin Chloridi...gr. xxiv.

Aq. Menthæ,............℥ vj.

S. ℥ ss, every 2 or 3 hours.

Benefit was apparent in twenty-four hours.—*Philadelphia Medical Times and Indiana Journal of Medicine.*

THE crayon or pure alum is employed by Magnus instead of copper sulphate in chronic and granular conjunctivitis. It is less painful, and the results are perhaps more durable. Frankel has extended its employment with satisfaction to the diseases of woman. It dissolves rapidly into the uterus without exciting uterine colic. It renders good service also in abscesses of scrofulous glands.—*Gaz. Hebdom from Bresl. Ærtzl. Zchft.*

**Nearsightedness and the Color of the Eyes.**—Mr. Nicate stated, at the meeting of the French Society for the Advancement of Science, that as one of the results of his examination of 3,434 eyes in relation to myopia, at Marseilles, this defect was observed far more frequently in light than in dark eyes, blue and gray eyes furnishing 18 per cent., and black and brown eyes only 11.27 per cent.—*The Hospital Gazette.*

